# How I approach weaning from venoarterial ECMO

**DOI:** 10.1186/s13054-020-03010-5

**Published:** 2020-06-08

**Authors:** Justin A. Fried, Amirali Masoumi, Koji Takeda, Daniel Brodie

**Affiliations:** 1 Department of Medicine, Division of Cardiology, Columbia University Medical Center/New York-Presbyterian Hospital, 622 West 168th Street, PH Building, 4th Floor, Room 129, New York, NY 10032 USA; 2 Department of Surgery, Columbia University Medical Center/New York-Presbyterian Hospital, New York, NY USA; 3 Division of Pulmonary, Allergy, and Critical Care Medicine, Department of Medicine, Columbia University Medical Center/New York-Presbyterian Hospital, New York, NY USA

**Keywords:** Venoarterial ECMO weaning, VA-ECMO weaning, ECMO weaning, Cardiogenic shock ECMO, Extracorporeal life support, ECLS

## Introduction

Venoarterial extracorporeal membrane oxygenation (VA-ECMO) is a temporary mechanical circulatory support device capable of providing robust cardiopulmonary support for days to weeks, most commonly in the setting of refractory cardiogenic shock or cardiac arrest as a bridge to recovery or heart replacement therapies (HRT, e.g., durable left ventricular assist device or cardiac transplantation) [1]. Given the complications associated with prolonged use of VA-ECMO, it is imperative to institute systematic weaning strategies. In this article, we review our standardized approach to VA-ECMO weaning in adult patients.

## Considerations prior to weaning

VA-ECMO supports circulation while treatment of the underlying pathology is prioritized to facilitate successful weaning or bridge to HRT. Because the potential for ventricular recovery is often difficult to ascertain, we advocate that all patients where HRT is a realistic consideration undergo early evaluation even if the intended goal is recovery [2–5].

Our center favors the use of invasive hemodynamics whenever possible to guide therapy in VA-ECMO. A pulmonary artery catheter provides valuable information about left ventricular (LV) loading conditions [6]. A right upper extremity arterial line should be maintained in all patients with femoral cannulation to facilitate monitoring of pulsatility and oxygenated blood flow to the arch of the aorta [7]. We generally target a cardiac index > 2.2 L/min/m^2^, mean arterial pressure (MAP) 65–80 mmHg, central venous pressure 8–12 mmHg, and pulmonary capillary wedge pressure < 18 mmHg. Arterial blood gas (ABG), lactate, hepatic, and renal function are followed in serial laboratory measurements to assess adequacy of end-organ perfusion.

In the event of concomitant use of a LV venting device [intra-aortic balloon pump (IABP) or percutaneous left ventricular assist device (pLVAD)], it is our approach to prioritize weaning and removing VA-ECMO first, given its propensity to increase afterload on a failing myocardium and relatively high complication rate [8]. One exception is when complications arise directly related to the ancillary device necessitating its removal. When used as a LV venting device, we maintain the IABP at 1:1 for unloading and ensuring adequate washing of the aortic root. Concomitant pLVADs, which in our institution is most often an Impella (Abiomed, Danvers, MA), are generally maintained at relatively low levels of flow (1.5–2 LPM) as its primary use in this setting is LV decompression rather than circulatory support [8–10].

**Fig. 1:**
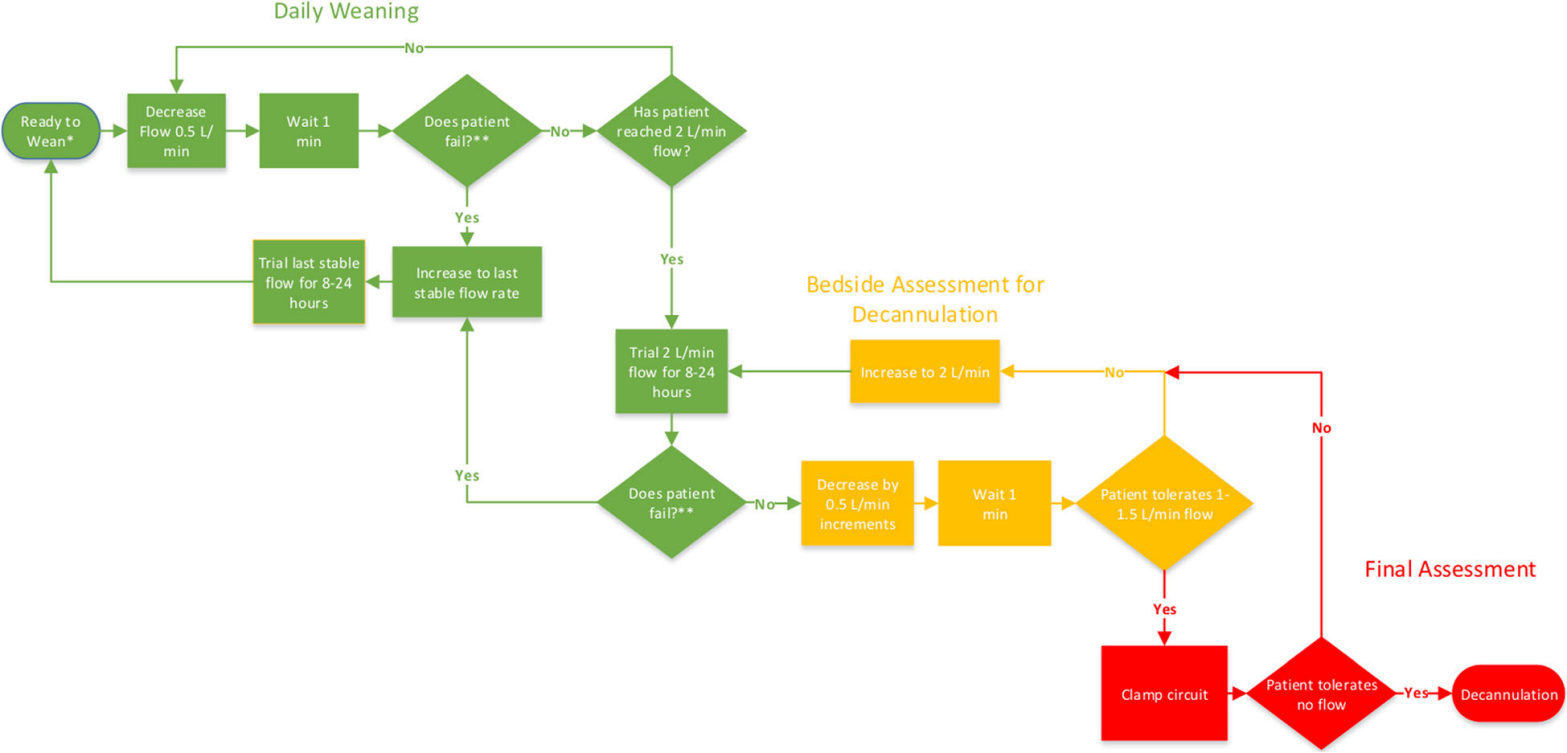
VA-ECMO weaning process. *Criteria required to initiate weaning trial: (1) Phenotype is compatible with recovery (2) End-organ function is improving (3) Pa02/Fi02 > 100 (4) Vasopressors and intropes at low levels (norepinephrine ≤ 4 μg/min, dobutamine < 5 mcg/kg/min) **Any of the following criteria constitutes failure of a weaning trial: (1) MAP falls below 65–70 mmHg or decreases by more than 10 mmHg from baseline (2) Significant increase in intracardiac filling pressures (3) Deterioration in respiratory status

Prior to weaning VA-ECMO flow, we wean vasoactive medications to low levels, given the deleterious effects associated with these medications, including arrythmia, renal injury, and limb ischemia. The following criteria should be met prior to weaning VA-ECMO: first, the patient phenotype is compatible with recovery; second, end-organ function is recovering; third, Pa0_2_/Fi0_2_ > 100; and fourth, vasopressors and inotropes are at reasonably low levels (for instance norepinephrine ≤ 4 μg/min or dobutamine < 5 mcg/kg/min). When these criteria are met, we initiate a 3-part approach to weaning that includes the following: (1) *daily weaning study*, (2) *bedside assessment for decannulation*, and (3) *final assessment* (Fig. 1).

## Daily weaning study

We perform daily transient reductions in ECMO flow rate in all patients to assess suitability for weaning, analogous to the strategy of daily spontaneous breathing trials in mechanically ventilated patients. We reduce flow rates in increments of 0.5 LPM. At each incremental drop in blood flow to a minimum of 2 LPM, we wait approximately 1 min to assess the effect on MAP and intracardiac pressures. If the MAP falls more than 10–15 mmHg or below 65 mmHg, the patient is not yet ready to wean to that level. Significant increases in right-sided filling pressures during weaning may also constitute failure especially in the setting of predominant right ventricular failure. At the end of the study, the blood flow rate is set at the lowest level achieved in the weaning study required to maintain stable MAP and intracardiac pressures. ABG, lactate, and a full set of invasive hemodynamics are obtained at this new flow rate to detect any impact on hemodynamics, tissue perfusion, or respiratory status [7]. Because a subset of patients who tolerate reduced support transiently are unable to tolerate the decreased support over a prolonged duration, lower flow rates are maintained for a minimum of 8 h prior to further weaning attempts. Weaning trials are performed at least every 24 h. Bedside echocardiography is used to provide additional information about native cardiac function as blood flow rates are reduced, particularly if the patient has failed prior weaning attempts. Prior literature has suggested the following parameters to be associated with successful weaning: aortic VTI ≥ 10 cm, LVEF > 20–25%, and lateral mitral annulus peak systolic velocity > 6 cm/s [11].

## Bedside assessment for decannulation

After a patient has tolerated a trial of 2 LPM for a minimum of 8 h with stable end-organ function, a bedside assessment for decannulation is performed. Flow is gradually decreased to 1 LPM over the course of approximately 1 min to detect hemodynamic instability with minimal ECMO support. If tolerated, blood flow is then returned to 2 LPM, and plans are made for decannulation if the underlying cause of initial decompensation has been sufficiently addressed such that liberation from VA-ECMO is possible. If the patient fails the bedside assessment, then flow is returned to 2 LPM with plans to reassess every 24 h.

## Final assessment

When a patient has tolerated a trial of 2 LPM of blood flow for a minimum of 8 h with stable hemodynamics and end-organ function and tolerates transient flow reduction to 1 LPM at the bedside, the patient is typically taken to the operating room for ECMO decannulation. The final assessment is performed at this time, wherein blood flows are gradually decreased and the cannulae are clamped; hemodynamic and ABG parameters are checked. Focused echocardiography is often undertaken to assess the impact of flow reduction on biventricular function. If acceptable, decannulation is performed.

## Conclusion

Weaning from VA-ECMO remains a challenging but critically important step in device management. The key is to balance minimizing complications associated with device support with the potential for hemodynamic deterioration if support is prematurely discontinued. We advocate for a standardized and systematic approach to weaning but also recognize that deviation from the above approach may be required in specific clinical scenarios.

## Data Availability

Not applicable

## References

[1] Guglin M, Zucker MJ, Bazan VM, Bozkurt B, El Banayosy A, Estep JD (2019). Venoarterial ECMO for adults: JACC scientific expert panel. J Am Coll Cardiol.

[2] Cheng YT, Garan AR, Sanchez J, Kurlansky P, Ando M, Cevasco M (2019). Midterm outcomes of bridge-to-recovery patients after short-term mechanical circulatory support. Ann Thorac Surg.

[3] Garan AR, Malick WA, Habal M, Topkara VK, Fried J, Masoumi A (2019). Predictors of survival for patients with acute decompensated heart failure requiring extra-corporeal membrane oxygenation therapy. ASAIO J.

[4] Schmidt M, Burrell A, Roberts L, Bailey M, Sheldrake J, Rycus PT (2015). Predicting survival after ECMO for refractory cardiogenic shock: the survival after veno-arterial-ECMO (SAVE)-score. Eur Heart J.

[5] Garan AR, Eckhardt C, Takeda K, Topkara VK, Clerkin K, Fried J (2018). Predictors of survival and ability to wean from short-term mechanical circulatory support device following acute myocardial infarction complicated by cardiogenic shock. Eur Heart J Acute Cardiovasc Care.

[6] Truby LK, Takeda K, Mauro C, Yuzefpolskaya M, Garan AR, Kirtane AJ (2017). Incidence and implications of left ventricular distention during venoarterial extracorporeal membrane oxygenation support. ASAIO J.

[7] Brodie D, Slutsky AS, Combes A (2019). Extracorporeal life support for adults with respiratory failure and related indications: a review. JAMA.

[8] Cevasco M, Takayama H, Ando M, Garan AR, Naka Y, Takeda K (2019). Left ventricular distension and venting strategies for patients on venoarterial extracorporeal membrane oxygenation. J Thorac Dis.

[9] Russo JJ, Aleksova N, Pitcher I, Couture E, Parlow S, Faraz M (2019). Left ventricular unloading during extracorporeal membrane oxygenation in patients with cardiogenic shock. J Am Coll Cardiol.

[10] Akanni OJ, Takeda K, Truby LK, Kurlansky PA, Chiuzan C, Han J (2019). EC-VAD: combined use of extracorporeal membrane oxygenation and percutaneous microaxial pump left ventricular assist device. ASAIO J.

[11] Aissaoui N, Luyt CE, Leprince P, Trouillet JL, Leger P, Pavie A (2011). Predictors of successful extracorporeal membrane oxygenation (ECMO) weaning after assistance for refractory cardiogenic shock. Intensive Care Med.

